# On the Nature, Signs, and Treatment of Childbed Fevers

**Published:** 1854-12

**Authors:** 


ART. VI.— *On the Nature, Signs, and Treatment of Childbed Fevers; in a Series of Letters addressed to the Students of his Class.* By CHAS. D. MEIGS, M. D., Prof. of Midwifery, etc., etc. Philadelphia: Blanchard and Lea. 1854.

It is not to be expected that the duties of the reviewer should be always pleasant. In the particular instance now on hand, it is especially unpleasant. Previous attempts at a fair and candid reviewal of the publications of Dr. Meigs have been received in a spirit of ill-temper which attributes personal motives to all expressions of dissent or adverse criticism. In a formal letter to the New Orleans Medical Journal, Dr. Meigs has chosen to place himself in the light of a persecuted individual, of whom, forsooth, the critics are jealous. He has impugned the motives and science of every man who differs from him, and assuming to be the chiefest oracle of Lucina, the chosen priest at her classic altar, he utters anathemas at the heretics who question his infallibility.

The consciousness that anything we may say adverse to the scientifie merits of Meigs on Childbed Fever, will be received in a like uncharitable spirit, makes the duty of the reviewer in this instance so disagreeable, that were the book merely a harmless one, we should announce it as on sale, and having thus discharged our obligations to the courteous and enterprising publishers, we should say no more about it. But it happens that it contains doctrines from which most intelligent practitioners must dissent, and which, received by the youthful physician as *ex cathedra,* are calculated to work the most disastrous results in the treatment of disease. We shall not, therefore, forego the duty of a careful review and protest.

The tone and style of this volume are the same which have rendered their author already notorious in his letters on the Diseases of Women, and other works. What we have previously characterized as "Carlylese run mad" forms the basis of the style, and its tone is one of somewhat too free assumption of authority, and offensive egotism. Were Dr. Meigs the man he conceives himself to be, it would not bring his manner within the limits of good taste. Still, one can pardon much in an originator. He who conceives new doctrines, or discovers new facts, is licensed to assert his claim somewhat loudly. But we are not aware that the author has even this excuse. We are not so old, or yet so learned as we hope to be. Much that has been written is yet unread by us, and it is quite possible that Dr. Meigs has some claims to dictatorship in midwifery other than the possession of his chair in the Jefferson School; but if so, it has escaped our notice. In the north he is only known as the opponent of chloroform, of the speculum, and of all new things generally. It is undoubtedly a good office and a worthy function, to guard the way against the entrance of hurtful innovations, but it is one usually consigned to the old fogies of the brotherhood. Our Philadelphia brethren are particularly good at this—to use "the gentle Elia's" witty expression, "they never do anything else."

The faults of style which have been so earnestly insisted upon by many reviewers, are not the most prominent errors in this volume. They should not, however, pass without notice.

In adopting a declamatory and stilted phraseology, the most learned author must necessarily sacrifice scientific accuracy and positive description, to the desire to make the story tell well. Declamation assumes the place of intelligible doctrine, and though it may contain nothing from which we positively dissent; yet even the truth is told in such a vague, uncertain manner, and is so beclouded by dandyisms of expression, that it is neither easy to arrive at the author's meaning, or to obtain from it any guide for our conduct. We instance the following paragraph from page 152:

"As the areas of phlogosis expand wider and farther from their original centers, greater and still more important mischiefs continue to be perpetrated within the abdomen, and the nervous mass reels and trembles under the load of irritations it can neither overcome, nor even resist. The sanguiferous and nervous reäctions that flow out from their great centers, serve, not to cure, but only to augment the mischief, and draw on the ultimate ruin both of the organs and the centers, which fail, and faint, and die, beneath the exciting power of the topical lesions."

Is this pathology? Or is it what they call at the west "high-falutin'?"

Enough on this matter—turn we to doctrines.

The opinions of Dr. Meigs on the treatment of puerperal fever, may be gathered from the following passages:

"If you will but be bold, resolute, resolved, as Gordon charges you to be, you will overlook the whole scene, and perceive that the heads of the monster are cut off at one blow; or that, as Legonais expresses it, one head retains its vitality and will arise more terrible than before. Leave off with 8, 12, or 16 ounces abstracted, and you may go away from the bedside saying

'She will surely die;' but if you will courageously and resolutely persevere until twenty-four or more ounces, not too many more, are taken away, you may retire after your ministration, feeling assured that the duty is well done, and believing that the life of the patient will be saved. If she should not be rescued, it would not be because you had not fulfilled your professional duty as to this, the most important part thereof."—Pages 256–7.

*Rush redivivus!* The author treats the loss of the vital fluid in a woman already enfeebled by labor and its flowings, as a matter of no moment. The typhoid tendency — the tendency toward death — of these cases, is entirely disregarded. We must bleed, he says, *ad juyulare febrim;* must so reduce the "powers of phlogosis," that its "areas" shall no longer expand; in a word, we must cut short the inflammation. That is what every practitioner is most anxious to do, but will the letting of 12, 16, or 24 ounces of blood do it? So far as we may judge from an experience, fortunately limited, it will not. The same amount of bloodletting will not cut short a conjunctivitis. The student of modern pathology need not be told that inflammation does not depend upon the impulse of the heart for its existence. We regard this doctrine of depletion in puerperal fever, as a most unfortunate and dangerous error. The experience of all countries condemns it. We need only refer to the pages of our last issue for an account of an epidemic of erysipelatous peritonitis, in which the practice of bloodletting was followed by results which, had they made the same impression upon the reporters that their statement does upon other minds, would have surely led to a search for some other means of cure.

In speaking of the use of cathartics, Dr. Meigs entertains, to a certain extent, the same views which have now become so general in the profession. At the same time, he, while discouraging their use, does not assert so strongly their mischievous qualities, as to make any impression upon the mind of the reader. Speaking of their use in purging off supposed impurities, he says:

"I seem to discern in them the dim and ghostly shadows of the defunct milk-metastasis dogma, wandering abroad half seen, half hid in a humoralism not less pernicious in its practical influences than the notions of *errement de lait*, coctions and lentors — * * * * * * *.

"All such morbid entities should be treated as tails of the lost sheep in the nursery rhyme:

'Little Bo-peep
Has lost his sheep,
And knows not where to find 'em;
Let 'em alone,

And they 'll come home,
Bringing their tails behind 'em.' "—Page 259, *op cit.*

But, to avoid great tympanitis, the learned author would give, grs. x., of calomel, at the commencement of the disease; and furthermore, in consideration of the tendency to distention from meteorism, he would frequently prescribe aperients: manna, carb. magnesia, &c.

A few pages back he has expressed his conviction that the operation of a purge can remove no *materies morbi*, and on page 302 he assigns as his reason for giving them, the existence of meteorism. An enormous tympanitis we recently observed in a case of choleraic dysentery, should, upon this hypothesis, have been removed by the repeated and copious alvine evacuations which occurred. We see the same feature in the diarrhœa of typhoid fever, but it does not often happen that a tympanitis is much, or permanently relieved by a purgation under any circumstances. If the meteorism is the only reason for Dr. Meigs' purgatives, would it not be as well to withhold them? For assuredly the constant perturbation and friction of inflamed surfaces, implied in the action of a cathartic, is not a trifling danger. Rest is a more powerful remedy in peritonitis than any bloodlettings.

Opium, which in the minds of many thoughtful men, assumes the highest importance in the treatment of peritonitis, receives such mention, and obtains such remedial credit, as may have been anticipated from our previous segregations.

In Dr. Meigs' theory, it is a remedy of trivial value, an adjuvant, to assist or modify the other means. He cautions his class against the use of a teaspoonful of laudanum per enema, and ridicules Dr. Kelly's cases of puerperal fever treated by what have been foolishly called "heroic doses" of opium.

This carries us far enough. If the treatment thus advocated by Dr. Meigs is the best now attainable, then is the science of medicine, in this respect, just where it was an hundred years ago. We have searched this volume in vain for a new suggestion of any importance; we find but the wearisome repetition of Dr. Gordon's aphorisms, the treatment of Sydenham and Rush, and for aught we know, of Paracelsus and Galen. In it is entirely neglected the great indication of *rest;* the faith of the practitioner is taught to rely on the doubtful remedy of bloodletting, and the constant perturbation of an inflamed surface by cathartics.

The "opium treatment," so scantily noticed, and so lightly valued by Dr. Meigs, rests upon a chain of reasoning which demands for it a more mature consideration. The experience of all men has gone to show that under bloodletting but a small ratio of cases recover. It, then, is unsatisfactory. The cathartic treatment, if it possesses any value, rests its claims upon the hypothesis of a *materies morbi* to be removed by purgation; and it is evident that it must necessarily do injury to the inflamed tissues themselves.

To meet the hiatus thus formed in other plans, the opium treatment has been devised. It has long been known that a full opiate will cut short an incipient pleurisy, as often as will a full bleeding. The inference derived from this has been successfully applied to an incipient peritonitis. Again, when the patient is kept semi-narcotized, the nervous system is spared that shock upon which Dr. Meigs insists so largely; its sensibilities are obtunded. The inflamed surface is kept in a position of entire rest, so that it may profit by topical means in the form of fomentations, or counter-irritation, and, as we have seen in repeated instances, the patient sleeps through a dangerous illness, to pass on to a recovery as rapid as that after bleeding is tedious and prolonged. The disturbance of cathartics is avoided, and patients do not suffer remarkably from tympanitis, even though there be no alvine evacuation for ten days together. Such is the more recent and more philosophical treatment which advancing minds would substitute for the antiquated notions of the volume under review; and, holding such opinions, we should have done less than justice to our convictions, had we withheld a free and hearty censure of the doctrines of Meigs on Childbed Fever.

We have made no allusion to a very loose pathology which pervades this work. All forms of child-bed fever are linked together as one disease, and the student who relied upon this apparently elaborate work for his distinctive diagnosis of metritis, pyæmia, and peritonitis, would find himself frequently at a loss. The distinctions of treatment founded upon these differences are equally important, and he who bled a peritonitis in accordance with the doctrines here inculcated, might be led by his success to do the same thing in a metritis with a very different result.

For sale by MILLER, ORTON & MULLIGAN. H.

